# Metal fused filament fabrication of the nickel-base superalloy IN 718

**DOI:** 10.1007/s10853-022-06937-y

**Published:** 2022-02-03

**Authors:** Yvonne Thompson, Kai Zissel, Andreas Förner, Joamin Gonzalez-Gutierrez, Christian Kukla, Steffen Neumeier, Peter Felfer

**Affiliations:** 1 Department of Materials Science and Engineering, Friedrich-Alexander-Universität Erlangen-Nürnberg, Institute I, Martensstraße 5, 91058 Erlangen, Germany; 2 Department of Polymer Engineering and Science, Institute of Polymer Processing, Montanuniversitaet Leoben, Otto Gloeckel-Str. 2, 8700 Leoben, Austria; 3 Materials Research and Technology Department, Luxembourg Institute of Science and Technology, Functional Polymers Unit, 5 rue Bommel, 4940 Hautcharage, Luxembourg; 4 Industrial Liaison Department, Montanuniversitaet Leoben, Peter Tunner Str. 27, 8700 Leoben, Austria

## Abstract

This study demonstrates metal fused filament fabrication (MF^3^) as an alternative additive and highly flexible manufacturing method for free-form fabrication of high-performance alloys. This novel processing, which is similar to Metal injection molding (MIM), enables a significant reduction in manufacturing costs for complex geometries, since expensive machining can be avoided. Utilizing existing equipment and reducing material expense, MF^3^ can pave the way for new and low-cost applications of IN 718, which were previously limited by high manufacturing costs. Iterative process optimization is used to find the most suitable MF^3^ process parameters. High relative density above 97% after pressureless sintering can be achieved if temperature profiles and atmospheres are well adjusted for thermal debinding and sintering. In this study, the influence of processing parameters on the resulting microstructure of MF^3^ IN 718 is investigated. Samples sintered in vacuum show coarse-grained microstructure with an area fraction of 0.36% NbC at grain boundaries. Morphology and composition of formed precipitates are analyzed using transmission electron microscopy and atom probe tomography. The γ/γ″/γ′ phases’ characteristics for IN 718 were identified. Conventional heat treatment is applied for further tailoring of mechanical properties like hardness, toughness and creep behavior. Fabricated samples achieve mechanical properties similar to MIM IN 718 presented in literature.

**Graphical abstract:**

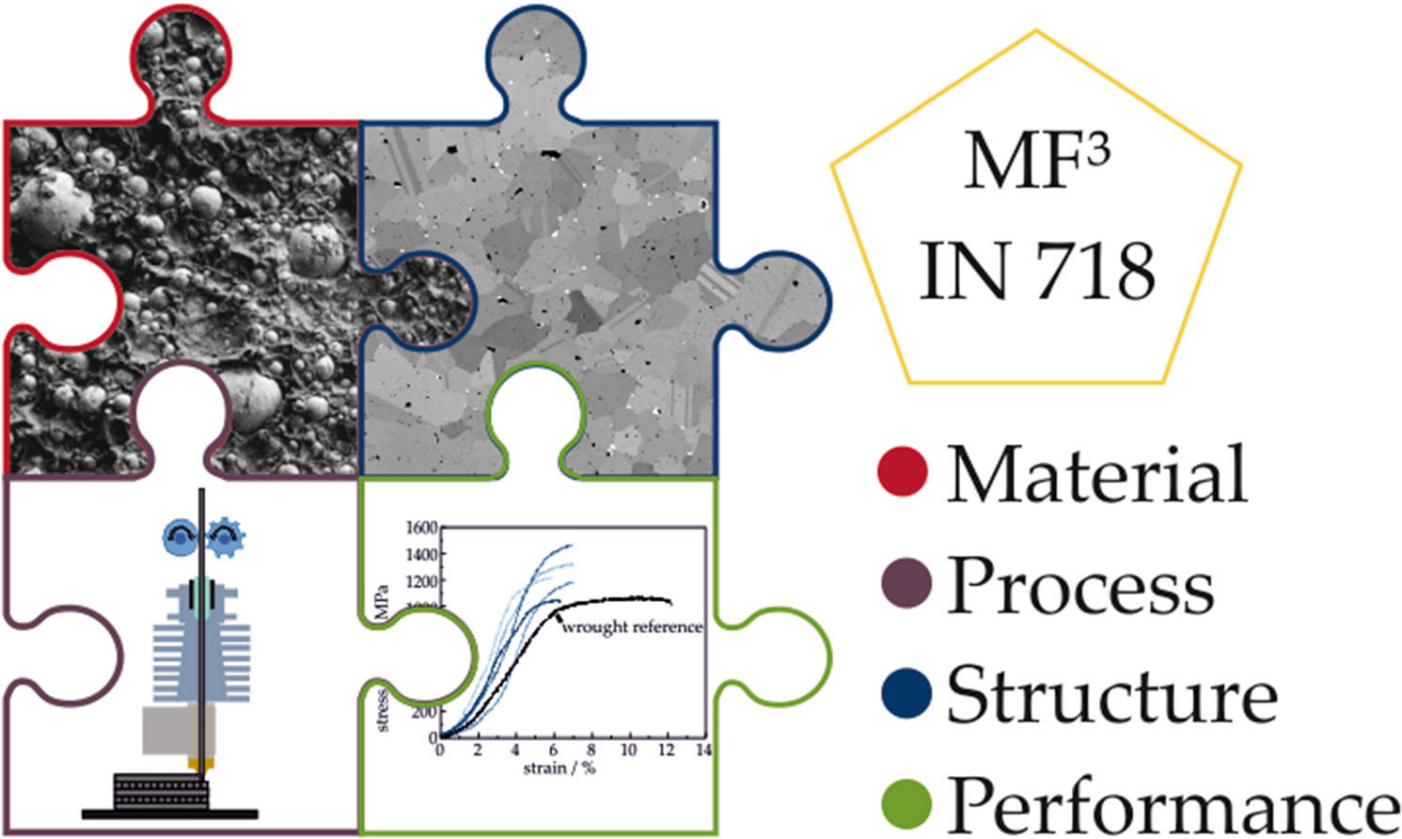

## Introduction

Nickel-base superalloys are widely applied in the aerospace and automotive industry due to their excellent mechanical properties and good corrosion resistance at high temperatures [1]. Conventional manufacturing comprises casting or forging followed by heat treatments for tailoring the desired microstructure and mechanical properties. To prevent formation of non-metallic inclusions (oxides, nitrides) during casting, a vacuum atmosphere is required, leading to high processing costs. Due to the high strength, toughness and hardness of nickel-base alloys, machining of complex geometries from wrought material is very cost intensive. Thus, metal injection molding (MIM) is used as a near-net shape complementary and alternative manufacturing technology for superalloy parts in large quantities [2–4]. IN 718 is the most used and investigated nickel-base superalloy and currently the only one with an established AMS (Aerospace Material Standard) for MIM production [5]. Heat-treated IN 718 is strengthened by finely dispersed γ″ (Ni_3_Nb) and γ′ (Ni_3_(Al, Ti)) intermetallic precipitates. The applied heat treatment consists of solution annealing followed by aging [6], which is adopted from existing cast and wrought material specifications. After heat treatment of sintered MIM IN 718, yield and tensile strength are increased and similar to cast material [7]. Hot isostatic pressing (HIP) after the sintering step can further increase ductility at a comparable tensile strength [8]. Sidambe et al. demonstrated that an optimized MIM process can achieve elongations that fulfill the requirements of AMS 5917 [5, 9]. In this case, the HIP step can be eliminated for further cost reduction.

The metal fused filament fabrication (MF^3^) has been investigated as an extension of the MIM process for free-form fabrication for a variety of metals [10–14]. During shaping, a green body is produced consisting of IN 718 powder particles and an organic binder. The green body is then subjected to different processes to remove the binder system and increase the inter-particle strength inside the remaining powder compact. The binder removal process is not 100% efficient in removing the organic components, and since there might be oxygen in the debinding atmosphere, carbon and oxygen residues are left in the debound and pre-sintered specimen. For successful MF^3^ processing of nickel-base superalloys, controlling the furnace atmosphere is key to reduce impurity uptake. Additionally, special focus is required on finding an appropriate temperature profile for thermal debinding and sintering. Heat treatment after sintering is applied for adjusting the resulting microstructure and enhancing mechanical properties. Knowledge acquired from investigations on MIM IN 718 [5–12, 15–17] can be applied for optimization of sintering parameters for MF^3^ processing.

Several atmospheres have been investigated for thermal debinding and sintering of MIM IN 718 [7, 9, 15, 16]. Highest densities were obtained by processing in vacuum at a temperature range of 1260–1290 °C that allows for liquid phase sintering [17]. The wetting liquid phase provides a capillary force that generates particle rearrangements and pore elimination, resulting in small pore sizes and low pore volume fraction [16]. The solidus and liquidus temperatures of fine powders are lower than the respective temperatures of wrought material due to their high surface energies and formation of oxides on particle surfaces [7]. For IN 718 powder with an average particle size of 13.1 µm, the solidus and liquidus temperature were identified to be 1260 °C and 1320 °C, respectively [16]. It is assumed that during sintering above the solidus temperature, liquid phases are formed by the eutectic of the γ′ phase, or by MC-type carbides and oxides at the grain boundaries [7]. This indicates increased liquid phase formation during sintering of MF^3^ processed IN 718, since an increased carbon content emerging from binder degradation and oxidation resulting from atmospheric oxygen is common for MF^3^ parts [14]. While some extent of a liquid phase enhances densification, excessive liquid phase must be avoided to maintain shape stability throughout the process. The powder specific sintering temperature is usually evaluated experimentally, and any previous processing history of particles and green bodies should be considered.

Sintering parameters have a major influence on the microstructure and properties of the final component. In most cases, the use of fine starting powders leads to fine grain structures of MIM IN 718 with mechanical properties similar to cast and solution treated material. Globular and fine microstructures with grain sizes of 10–30 µm were measured for IN 718 in as-sintered state after MIM processing [7, 18]. Additionally, the pick-up of impurities during debinding and sintering can result in the formation of carbides and oxides, often in form of prior particle boundaries (PPB) [2]. Oxides at grain boundaries hinder grain growth and further contribute to a fine-grained microstructure. An acceptable concentration of grain boundary carbides can improve high-temperature strength. However, extensive amounts of high-temperature stable carbides can act as crack initiation sites, harming mechanical properties [19]. Besides controlling the atmosphere to reduce carbon and oxygen uptake, grain growth can be regulated by sintering temperature and sintering time. If creep resistance is required, coarse grains are beneficial for high-temperature applications. Furthermore, size and morphology of carbides and other precipitates are influenced by heating and cooling rates during sintering [2].

The presented study investigates MF^3^ as a low-cost additive manufacturing (AM) method for IN 718 parts as opposed to powder bed fusion (PBF) or direct energy deposition (DED) methods that have been investigated by other researchers [20–22]. In this study, the influence of the debinding atmosphere on the resulting density and microstructure is compared for air, argon atmosphere and vacuum. Sintering was conducted in vacuum, and the sintering temperature was adjusted to achieve highest density at limited grain growth. The obtained samples were subjected to a standard heat treatment, and hardness measurements were used to indirectly confirm precipitation of γ′ and γ″ during aging. For further characterization of mechanical properties, meso-scale tensile testing was conducted at room temperature and high-temperature performance was investigated by creep testing. Detailed investigation on the evolution of the microstructure was conducted by transmission electron microscopy (TEM), and the final heat-treated state was studied by atom probe tomography (APT).

## Materials and methods

### Materials

IN 718 powder was incorporated into a previously developed multi-component binder system [10, 11] enabling metal fused filament fabrication. A maximum particle size of 25 µm was chosen to allow high powder loading and good extrudability. Particle size data according to laser diffraction analysis were provided as D_10_ = 3.4 µm, D_50_ = 8.5 µm and D_90_ = 25.7 µm. Nominal chemical composition of the powder is listed in Table 1 according to the supplier (Sandvik Osprey Ltd., GB). The chemical analysis was done by inductively coupled plasma atomic emission spectroscopy (ICP-OES), combustion analysis and glow discharge mass spectrometry (GD-MS). Contents of C and O are included due to their relevance for the MF^3^ processing.

**Table 1:** Chemical composition of the powder according to supplier information (values in wt%)

Ni	Cr	Fe	Nb	Mo	Ti	Al	C	O
51.5	18.8	Bal	4.98	3.1	0.77	0.2	0.015	N.A

**Figure 1:**
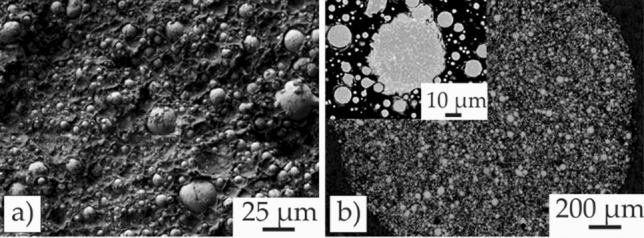
SEM micrographs, showing **a** filament fracture surface with particles embedded into the matrix of the polymeric binder system. **b** Cross-section of particles inside the filament. Little amounts of small spherical pores are visible inside the particles

To reduce shrinkage during thermal debinding and sintering, a high powder content of 55 vol% was selected for filament fabrication. The scanning electron microscopy (SEM) images displayed in Fig. 1a prove a homogeneous dispersion of particles inside the polymeric binder matrix. The customized binder system consists of two main organic compounds: a thermoplastic elastomer (Kraiburg TPE GmbH & Co. KG, Germany), which provides mechanical flexibility, and a grafted polyolefin (BYK Chemie GmbH, Germany), which adds stiffness. This combination is essential to unspool and reliably feed the filament to the extrusion unit without breaking or buckling. The binder system was developed for solvent debinding. Therefore, the thermoplastic elastomer is soluble in cyclohexane, and the grafted polyolefin is insoluble. The grafted polyolefin provides shape retention after the solvent extraction of the thermoplastic elastomer. The binder system was prepared by compounding two polymers in a co-rotating twin-screw extruder (ZSE 18 HP-48D Leistritz Extrusionstechnik GmbH, Germany). After compounding and pelletizing the binder, the IN 718 powder was added in a second compounding step in the same twin-screw extruder. The hopper of the compounder was firstly flushed with argon gas to reduce IN 718 oxidation. Finally, feedstock filaments were produced in a single screw extruder (FT-E20T-MP-IS, Dr. Collin GmbH, Germany). A detailed process description is provided in [13, 14, 23].

Thermogravimetric analysis (TGA) of the feedstock material implies a total binder polymer amount of 9 wt%. Thus, measurements of the removed polymer weight indicate an IN 718 powder content of 53.4 vol%. The volume fraction analysis was complemented using digital image correlation software Image J, which indicates a powder fraction of 51 vol% within the filament cross-section (Fig. 1b). Therefore, the actual powder content lies between 51 vol% and 53.4 vol%, which is lower than the originally planned content of 55 vol% due to feeding errors during the feedstock compounding process.

Cross-sectional images of the filament (Fig. 1) show the internal microstructure of the individual particles. Depending on the particle size, a cellular to columnar solidification morphology is developed as a result of gas atomization. Additionally, little amounts of spherical pores are visible that remain inside the material during the entire MF^3^ process due to their small size.

### Shaping

Shaping was realized by combination of 3D fused filament printing and subsequent green body compression to eliminate minor printing defects. This procedure was established in a previous investigation and proved successful for shaping of low-defect green parts [14].

A desktop 3D printer, Prusa i3 MK2 (Prusa Research, Prague, Czech Republic), served for 3D printing of the IN 718-loaded filament. A nozzle temperature of 280 °C allowed for continuous extrusion through a 0.6 mm diameter hardened steel nozzle. The nozzle diameter and the layer thickness will determine the resulting surface roughness of the manufactured part. Smaller nozzles than 0.6 mm tend to clog faster than larger ones; therefore, if a smoother surface is needed, post-printing operations such as laser polishing or other mechanical methods could be used in the green part before sintering [24, 25]. Completely dense infill was used with alternating rectilinear and concentric infill patterns. A slight over-extrusion was created by adjusting the “extrusion multiplier” to 1.2, extruding 120% of the geometrically necessary material volume. This eliminates minor printing gaps and ensures an overall dense infill, which helps to improve the mechanical properties of the printed specimen [26]. During the whole printing process, a maximum volumetric flow rate of 25 mm^3^ s^−1^ was used with a printing speed of 10 mm s^−1^. All printing parameters were set within the slicer software Simplify3D (version 3.1.1, Simplify3D, Cincinnati, OH, USA).

As previously reported [14], a cylinder hot mounting press (LaboPress 1, Struers, Copenhagen, Denmark) was used for densification of printed samples in green state. Within this study, the diameter of the cylindrical samples was defined by the dimensions of the cylinder press. A diameter of 23.5 mm and a printed height of 6 mm led to compressed samples with a height of 5.6 mm. Minor printing gaps were eliminated during pressing at 180 °C for 10 min at a pressure of 92 MPa. In the case of parts with complex geometry, warm-isostatic compression of the green body could be achieved by using liquids or gases, as presented by Paramore et al. [27].

Prior to debinding and sintering tests, the compressed green bodies were divided into four equal pieces, giving comparable parts for further evaluation of process parameters. The hourglass-shaped specimens for meso-scale tensile testing and cylinders for creep testing were fabricated by milling (Pocket NC V2-10, Bozeman, MT, USA) in green body state. To account for shrinkage during debinding and sintering, specimens were milled with an oversize of 25% in linear dimensions. Milling the test geometry from cylindrical green bodies allows testing of representative sample volumes. Thus, the influence of defects at the edges of printed parts that can lead to premature failure as observed in [11] was minimized.

### Debinding

Removal of organic compounds is realized in a two-step debinding process. Preliminary solvent debinding removes the main polymer content and enables accelerated thermal debinding. During the first debinding step, cyclohexane (≥ 99.5%, Carl Roth GmbH + Co KG, Karlsruhe, Germany) is used to dissolve and chemically extract the soluble fraction (i.e., TPE). While solvent debinding progresses from the surface into the inner regions of the green body, pore channels are opened as polymer subsequently diffuses out of the body. A total of 98.5 wt% of the theoretical soluble content must be removed to create sufficient pathways for the evaporation of degradation products during thermal debinding [23]. The success of the solvent debinding process was checked by measuring the weight loss. It was considered complete as soon as at least 98.5 wt% of the contained soluble component was removed.

Afterward, the remaining insoluble backbone polymer (i.e., polyolefin) must be thermally degraded at elevated temperatures. Different atmospheres were tested during this step to investigate best conditions for removal of degradation products while conserving the sintering activity. Argon flow (Argon 4.6, Rießner Gase GmbH, 99,996% Ar) was set to 50 ml min^−1^, while the vacuum pressure varied between 10^–3^ and 10^–5^ mbar, depending on the amount of volatilized binder. Knowledge gained from the TGA measurement and previous studies [10, 14] was used to define the appropriate temperature range. After additional experimental studies, a stepwise heating program with a minimal rate of 0.1 °C/min between 170 and 550 °C was defined for thermal debinding without the risk of defect formation. For details on the choice of process parameters, see Sect. “Debinding” in the results part.

### Sintering and heat treatment

Sintering of the completely debound parts was performed in vacuum at 10^–4^ mbar. Different sintering temperatures between 1260 and 1320 °C were tested aiming at highest densification. Sintered samples were characterized by Archimedes density measurements and microstructural analysis using backscattered electron (BSE) imaging and electron dispersive X-ray spectroscopy (EDS) at an accelerating voltage of 20 kV with a 120 µm aperture (Zeiss Crossbeam 1540, Carl Zeiss Microscopy GmbH, Jena, Germany). Based on these investigations, an optimal sintering program with a holding time of 4 h at 1280 °C was used for the preparation of specimens for further investigations (Sect. “Sintering”). A typical heat treatment consisting of solution annealing at 980 °C for 1 h followed by air cooling with subsequent aging at 720 °C for 8 h and 620 °C for 8 h in air was applied to create a tailored microstructure with γ′ and γ″ precipitates. The purpose of solution annealing is to homogenize the Nb-content and increase the availability of Nb for precipitation of γ″. The subsequent two-stage aging treatment contributes to precipitation of optimized phase fractions of γ′ and γ″. To investigate the γ′ precipitate fraction and morphology, foils of 200 µm thickness were cut, ground, electrolytically thinned (A3, Struers GmbH, Germany) and characterized using a *Philips CM200* transmission electron microscope (TEM) at a high voltage of 200 kV. The volume fraction of MC-type carbides was determined by image processing of SEM images. A total of 8 frames were binarized and analyzed to calculate the mean volume content.

APT experiments were carried out in a CAMECA LEAP 4000X HR (CAMECA Inc. Madison, WI, USA) using laser mode with a pulse energy of 50 pJ and a pulse rate of 200 kHz to trigger field evaporation at 1% of the pulses. The base temperature was set to 50 K. Data processing was done in IVAS (Cameca Inc. Madison, WI, USA) and MatLab (MathWorks Inc., Natick, MA, USA) with a custom algorithm developed in-house [28]. Chemical analysis by spark spectrometry was done with SpectroMaxx 06 (SPECTRO Analytical Instruments GmbH, Kleve, Germany) on ground cross-sections under argon flow.

### Mechanical characterization

To evaluate the success of the heat treatment, Vickers hardness of as-sintered, solution-annealed and heat-treated specimens was measured according to DIN EN 6507 (HV0.2/14). A small load of 1.96 N was chosen to enable the placement of adjacent indents along the cross-section. A total of 20 indents were averaged for each measured sample. Further mechanical characterization of heat-treated specimen was realized by micro-tensile testing (Kammrath & Weiss GmbH, Dortmund, Germany) and creep tests (custom-build pneumatic device). The hourglass-shaped specimens for micro-tensile testing were cut to a thickness of 0.5 mm after sintering. With a minimal width of 1 mm, the resulting effective cross-sectional area during tensile testing is 0.5 mm^2^. Tests were controlled by a constant displacement of 5 µm s^−1^. Creep specimens had a cylindrical shape with a diameter of 4 mm and a length of 6 mm. Tests were performed on aged samples at 650 °C in air under constant compression stresses between 650 and 800 MPa.

## Results and discussion

### Shaping

Fused filament fabrication (FFF) of the highly loaded filaments can be realized on a simple desktop 3D printer without major adjustments. The 0.6 mm hardened steel nozzle allows for continuous extrusion at 280 °C. For overall consistent infill, alternating rectilinear and concentric infill patterns were used with an overlapping area of 20% of adjacent printed strands (corresponding to 20% *infill overlap*). The printing parameters were kept identical for all specimens investigated in this study according to Table 2.

**Table 2:** Process parameters for FFF of IN 718-loaded filaments

Process parameter	Unit	Value
Printing speed	mm s^−1^	10
Nozzle temperature	°C	280
Bed temperature	°C	20
Layer height	mm	0.1
First layer height	mm	0.1
Extrusion multiplier		1.2
Infill overlap	%	20
Extrusion width	mm	0.75

Together with subsequent compression under 92 MPa for 10 min at 180 °C, additive shaping of defect-free green bodies was successfully realized.

### Debinding

**Figure 2:**
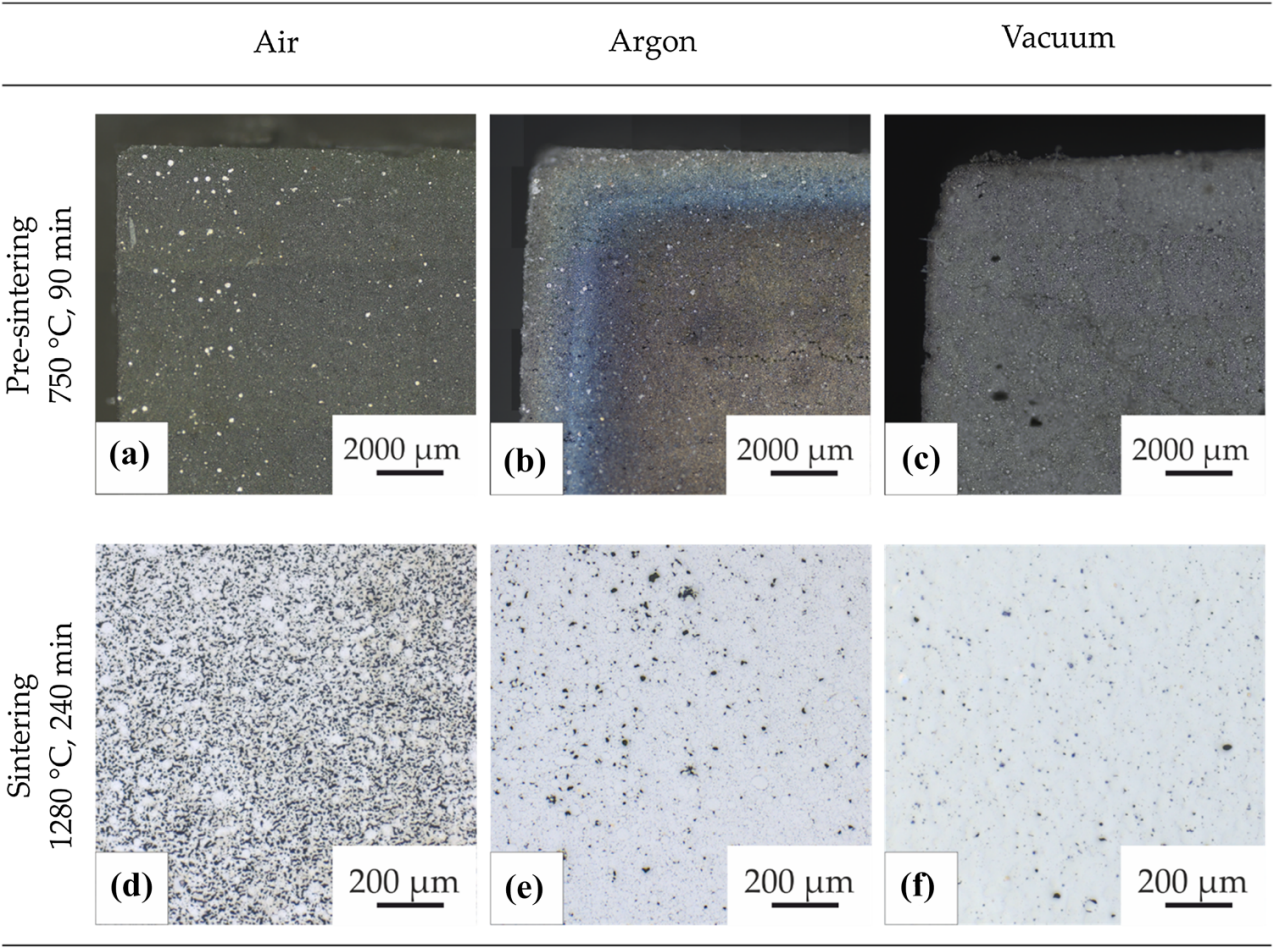
Cross-section of IN 718 samples after thermal debinding and pre-sintering in **a** air at 1 °C min^−1^, **b** argon atmosphere at 0.1 °C min^−1^ and **c** vacuum at 0.1 °C min^−1^. Bright spots and the light and blue appearance of the near-surface region in b) indicate oxide formation on IN 718 particles during exposure. Black spots in **d**–**f** show the respective remaining porosity after subsequent sintering in vacuum at 1280 °C for 240 min

Solvent debinding in cyclohexane for at least 7 days reliably removed the main binder component, achieving the required weight loss of 98.5 wt% of the soluble binder component. As previously reported, this duration can be reduced to 60 h by using 250 ml of fresh cyclohexane per 100 g sample mass [10]. Alternatively, recirculating debinding equipment typically used in the MIM industry can further accelerate the solvent debinding process. Sample integrity is high during extraction of the soluble binder component, and no defect formation was observed in any of the processed parts. To maintain particle cohesion during thermal degradation and removal of the remaining backbone polymer, adjusting of the heating rate is decisive, especially in the critical temperature range (170–550 °C). Experimental variation of heating rates revealed that a reduction to 0.1 °C min^−1^ was necessary to consequently prevent cracking or blistering. This rate needs to be applied between 170 and 550 °C to give enough time for binder degradation and evaporation. It is lower than applied heating rates for steel-loaded filaments with the same binder [10, 29]. The adapted heating rate is necessary for thermal debinding in atmospheres with low oxygen partial pressure (pO2), whereas debinding in air was successful also at higher rates up to 1.0 °C min^−1^ in this study. The thermal debinding step is completed by pre-sintering at 750 °C for 90 min to generate particle rearrangements and create enough stability to withstand sample handling. It is important to note that the suitable heating rates for thermal debinding strongly depend on the size and wall thickness of the part. Higher heating rates can be applied when reducing the wall thickness. Debound samples show notable weight loss that indicates the removal of the remaining organic binder components. Weight loss in vacuum is close to the theoretical amount of backbone polymer inside the green bodies. For samples debound in argon atmosphere, the weight loss is 28% lower than in vacuum. Finally thermal debinding in air leads to a weight loss 89% lower than in vacuum. In the latter case, the weight gain due to oxygen uptake from the furnace atmosphere levels out the weight loss caused by binder burnout. The cross-sections of samples after thermal debinding in air, argon atmosphere and vacuum are presented in Fig. 2.

After processing in argon atmosphere, a significant atmospheric influence along the sample rim is visible by light and blue appearance of the near-surface regions indicating oxygen uptake down to a depth of several hundred micrometers. This oxide formation on IN 718 particles is due to higher levels of remaining oxygen in argon atmosphere than in the applied vacuum levels of 10^–4^ mbar. Densities after pre-sintering for 90 min at 750 °C also vary significantly for different atmospheres. While 87.2 ± 2.8% of the theoretical density (TD) of IN 718 (TD = 8.2 g cm^−3^) was achieved for vacuum processed samples, pre-sintering in argon only led to a relative density of 79.9 ± 3.5%. In case of air processing, the oxygen uptake reduces the densification significantly, leading to a density of the partially debound and pre-sintered part of only 67.4 ± 4.7% TD. The inhibited densification is a result of oxidation on particle surfaces during thermal debinding. Oxides hinder diffusion and reduce sintering activity. Thus, more time at higher temperatures is necessary to achieve densification comparable to vacuum atmosphere. Nevertheless, stability of samples is high enough to allow handling after processing in air despite the lower density, as oxide scales on powder surfaces increase friction between particles resulting in better cohesion.

### Sintering

**Figure 3:**
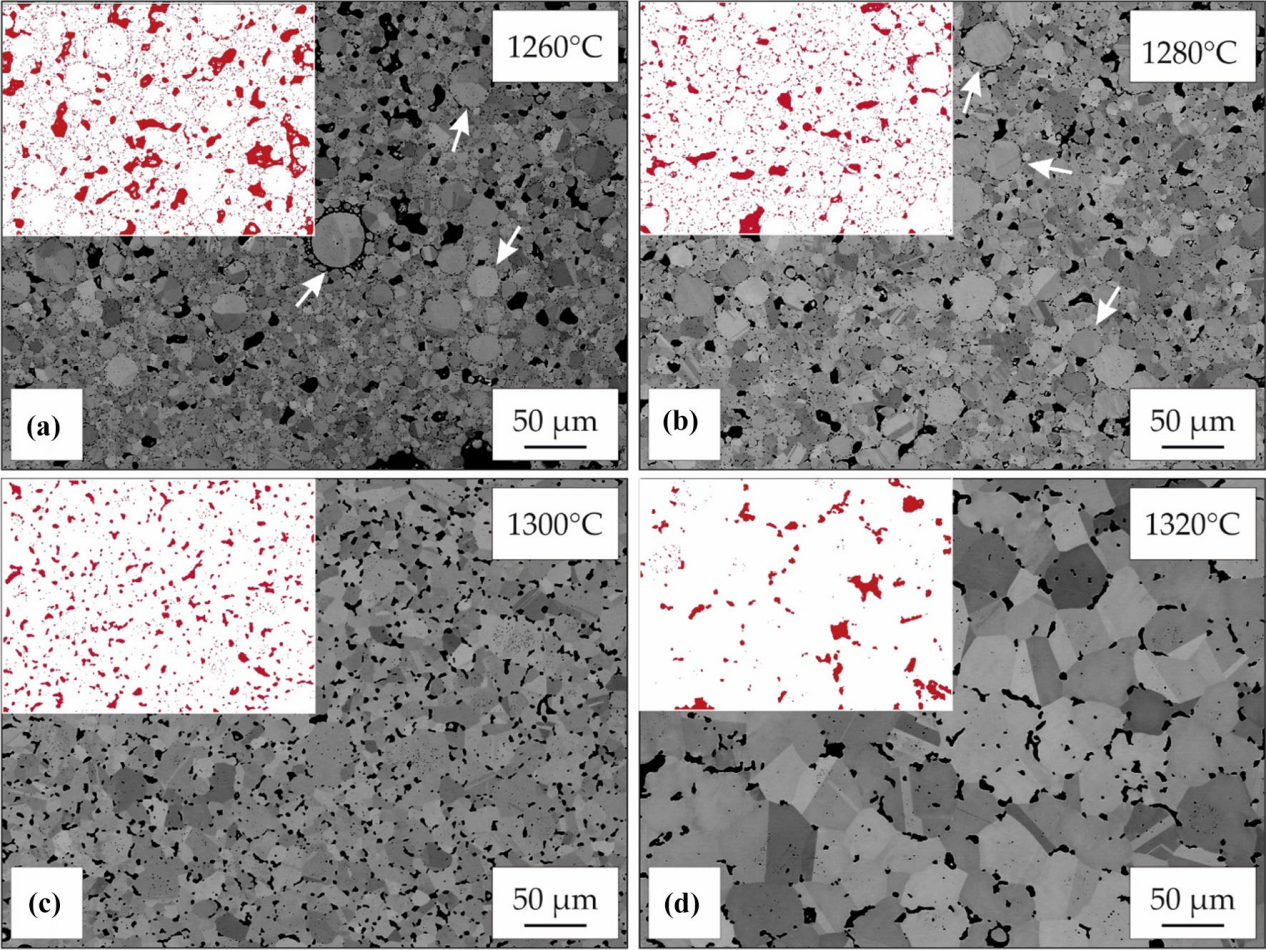
Microstructure evolution after debinding and pre-sintering in argon atmosphere with subsequent sintering in vacuum at** a** 1260 °C,** b** 1280 °C,** c** 1300 °C and** d** 1320 °C for 4 h. White arrows in** a** and** b** mark prior particle boundaries resulting from oxidation of particle surfaces during processing. Red regions in the binarized images mark the remaining porosity

Sintering of oxidized particles after debinding in air can hardly create any further densification. Resulting samples remain highly porous as shown in Fig. 2d implying that an oxygen containing atmosphere is not suitable for MF^3^ processing of IN 718. After argon and vacuum debinding, sintering in vacuum above 1260 °C creates pore elimination and relative densities above 90% (Fig. 2e, f). Since used temperatures lie above the solidus temperature, a liquid phase is formed, which enhances densification. Microstructure evolution after sintering in vacuum for 4 h at different sintering temperatures is displayed for samples debound under argon atmosphere in BSE images in Fig. 3. Porosity decreases with increasing sintering temperatures up to 1300 °C. At a higher temperature of 1320 °C, no further densification can be generated, but significant grain growth is visible. Higher temperatures than 1300 °C are not favorable, as shape loss occurs due to high amounts of liquid phase. Sintered samples show a high number of grain boundary pores as a result of oxidation during thermal debinding in argon. Oxides formed on particle surfaces hinder sintering in the further course of MF^3^ processing, resulting in an increased remaining porosity. Since pores cause grain boundary pinning [30–32], grain sizes remain small and grain coarsening only sets in when sintering near the liquidus temperature at 1320 °C. The achieved maximum density of sintered IN 718 after debinding in argon atmosphere is 94% TD.

**Figure 4:**
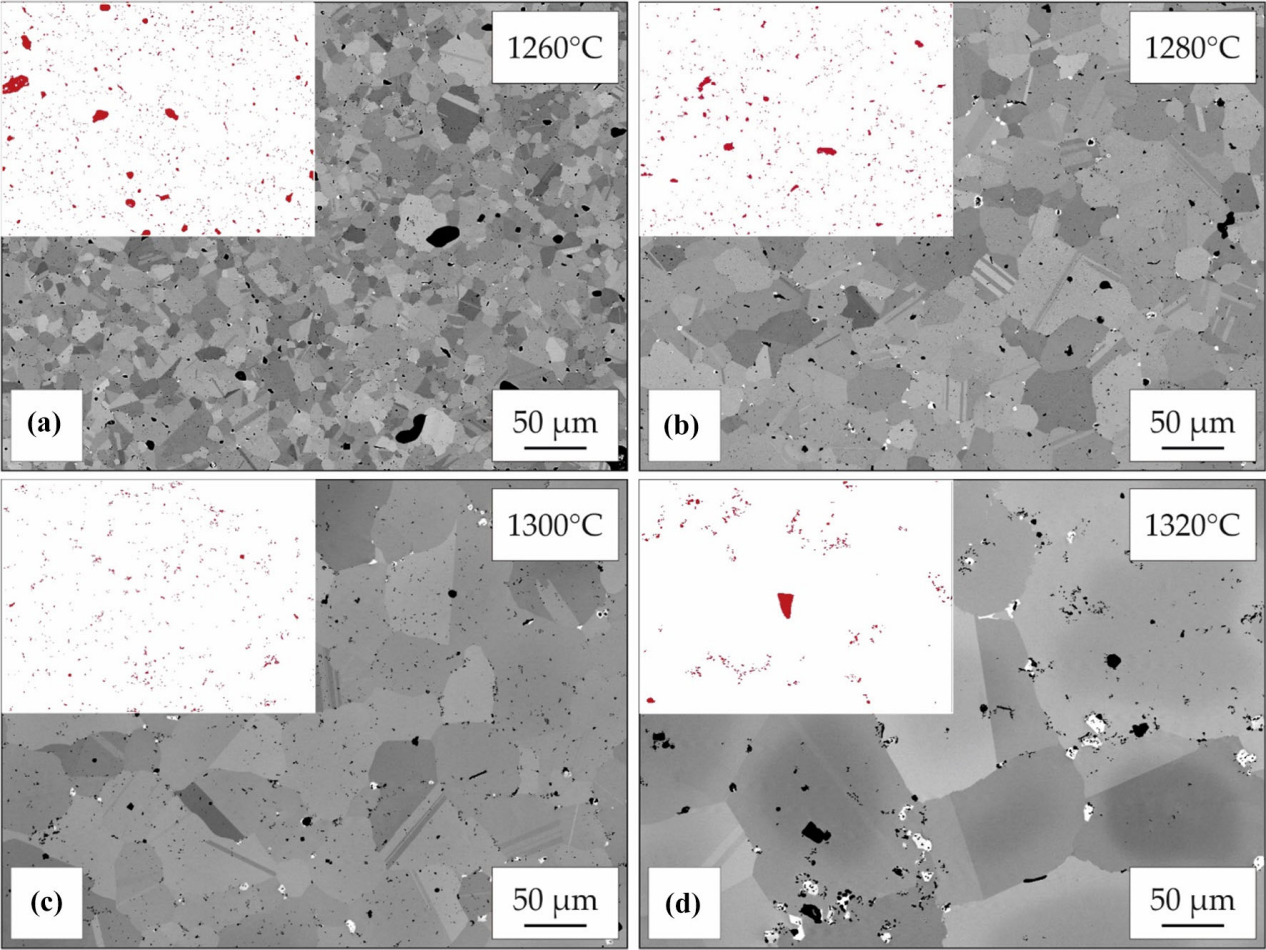
Microstructure evolution after debinding and sintering in vacuum at** a** 1260 °C,** b** 1280 °C,** c** 1300 °C and** d** 1320 °C for 4 h

In comparison, samples consistently processed in vacuum at levels of at least 10^–4^ mbar during debinding and sintering show little residual porosity at grain boundaries (Fig. 4). Pore elimination is enhanced by increasing temperatures up to 1280 °C. Strong grain coarsening is demonstrated above 1300 °C. The reduced grain boundary pinning in comparison with argon sintering is mainly attributed to the lower amount of grain boundary porosity. Phase fractions of other pinning phases (e.g., NbC and TiN) that were identified after vacuum debinding are at low levels compared to the amount of grain boundary porosity. The difference in grain coarsening between the two debinding atmospheres is therefore more likely attributed to the pinning effect of pores. The resulting microstructure of vacuum processed samples is coarse-grained. As grain boundaries break away from pores during grain growth, remaining pores are mainly isolated pores with a spherical shape located inside the grains. Best trade-off between densification, grain growth and geometrical stability is obtained by sintering at 1280 °C for 4 h. Relative densities above 97% were consistently achieved using theses parameters. The observed microstructure is in good agreement with the results of injection molded IN 718 as reported by Özgün et al. [7].

Samples sintered in vacuum show carbide formation at grain boundaries. EDS demonstrates enrichment of Nb in these phases together with decreased Ni concentrations. Thereby these phases were identified as NbC, which are common primary carbides in IN 718. The area fraction of these MC-type carbides is 0.36% and similar to values of wrought and heat-treated IN 718, implying that low process-related carbon uptake has taken place [33]. This indicates a good control of thermal debinding preventing carbon uptake emerging from binder degradation. While this is in contrast to the increased impurity uptake observed in literature [7], sintering at low temperatures after argon debinding leads to formation of oxides at PPBs (white arrows in Fig. 3a, b) in accordance with investigations on injection molded nickel-base superalloys [2]. Consequently, the covered particles exhibit no sintering activity up to temperatures of 1280 °C and show increased impurity uptake during processing.

As a result of the observed microstructural evolution, vacuum processing (1280 °C, 4 h) is favored to achieve higher densities of sintered parts. The associated grain coarsening is accepted and may even be beneficial for enhanced creep performance.

### Sintered and aged microstructure

**Figure 5:**
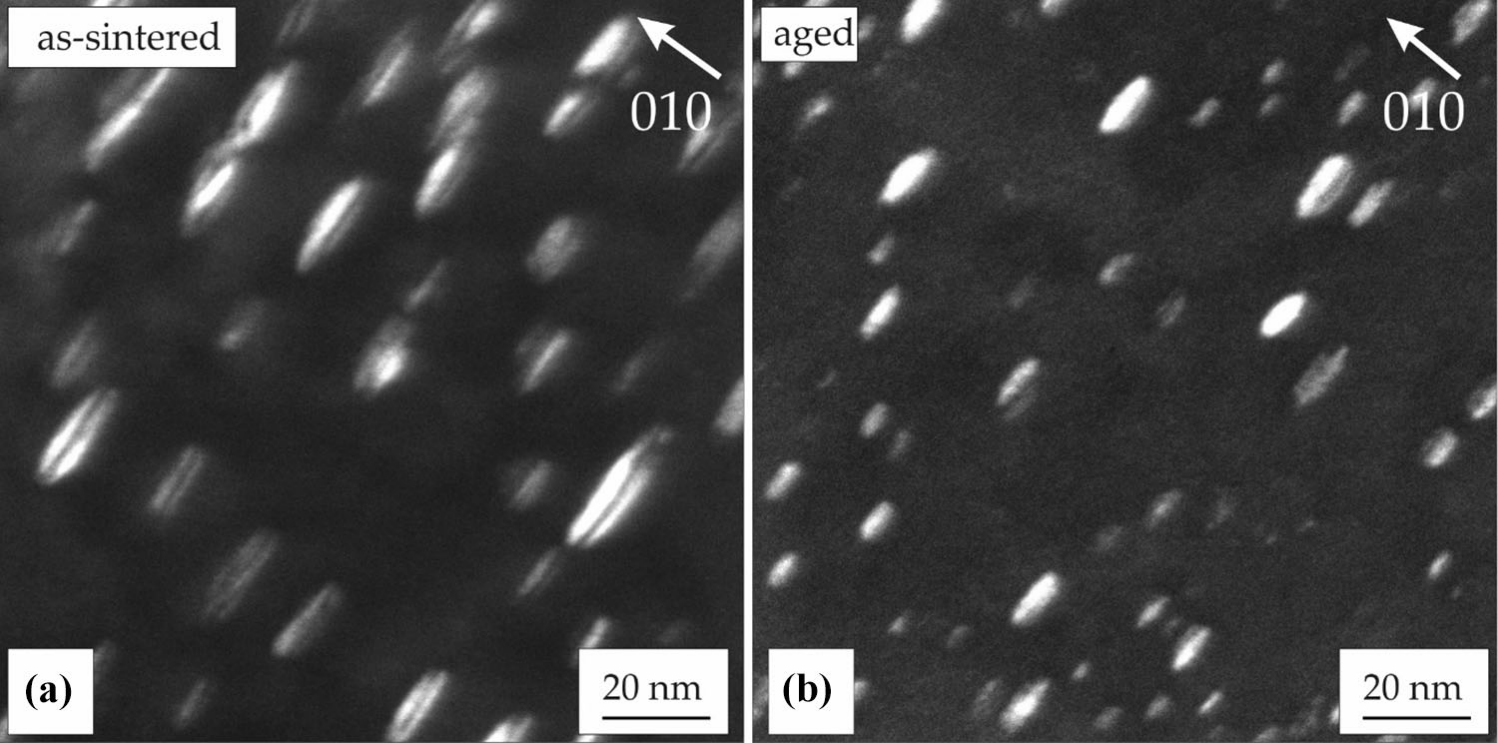
TEM micrographs of **a** as-sintered and** b** heat-treated IN 718 taken with **g = 010** (displaced aperture dark field) in two-beam conditions. **a** Precipitation of disk-like γ´/ γ″ co-precipitates already occurs during slow furnace cooling at 3 °C/min. **b** Finer disk-like γ″ and γ′/γ″ co-precipitates are achieved after aging

Sintered samples were subjected to a heat treatment that provides combined strength and ductility by creating an ordered microstructure with γ′ and γ″ precipitates. A standard heat treatment established for conventional IN 718 was applied, consisting of solution heat treatment with the subsequent two-step aging. As visible in the TEM images in Fig. 5, precipitates can already be found in the as-sintered state. These are mainly γ′/γ″ co-precipitates with disk-like shape. They form during the slow furnace cooling after sintering when the respective precipitation windows are crossed [34]. Solution annealing completely dissolves these precipitations and creates a supersaturated solid solution. Hardness measurements that are in detail presented in Sect. 3.5 demonstrate high hardness of the as-sintered state that is reduced by solution annealing. These results prove that rapid air cooling after solution annealing prevents time induced precipitation preserving the precipitate-free state with homogeneous distribution of Nb. Subsequent annealing at 720 °C and 620 °C for 8 h each deliberately allows γ′ and γ″ formation contributing to higher hardness and strength. Figure 5b shows a TEM image of the aged microstructure with finer disk-like γ″ precipitates. Additionally, some γ′/γ″ co-precipitates are visible [35–38].

**Figure 6:**
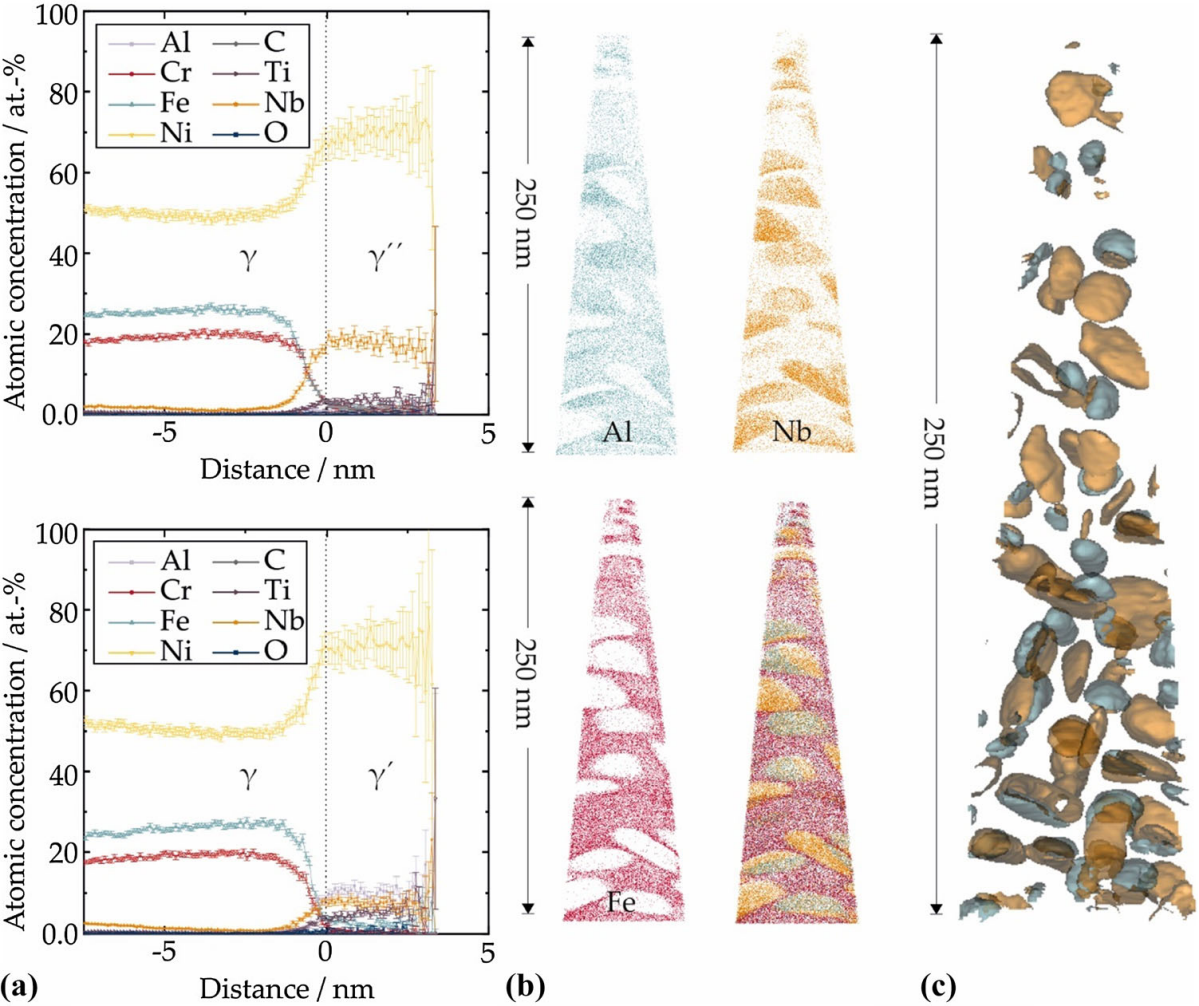
APT data of the sintered (vacuum, 1280 °C, 4 h) and heat-treated (air, 980 °C, 1 h; 720 °C, 8 h + 620 °C, 8 h) sample:** a** Proxigrams of elements showing the transition of matrix phase into the γ″ and the γ′ phase.** b** Ion distribution for the strongly partitioning elements Al, Nb and Fe.** c** Isosurfaces of 6.5 at% Al and 11.5 at% Nb representing the γ′ and γ″ precipitates respectively

The difference in chemical composition between the γ matrix and the γ′ and γ″ precipitates is confirmed by APT concentration profiles (Fig. 6b). Cr and Fe show a strong partitioning toward the γ matrix, whereas the γ″ phase shows a high concentration of Nb. The γ′ precipitates show enrichment of Al, Ti and Nb (Fig. 6a). Isosurfaces of Al (blue) and Nb (orange) visualize γ′ and γ″ precipitates inside the γ matrix (Fig. 6c). The determined chemical compositions of all three phases in this standard heat-treated condition are given in Table 3 and fit well with values of commercial Alloy 718 that was equally heat-treated [36]. Peak deconvolution based on isotopic abundances gives a reduction in the Cr content by 3 at% in comparison with the original powder composition measured by the supplier (given in Table 1). The decrease in Cr is due to evaporation during sintering in vacuum that has been observed in the same way during sintering of IN 718 before [39]. Additionally, the MF^3^ processing history leads to an increased amount of oxides, whereas the content of solute C is close to zero despite using an organic binder during shaping. Hence, APT demonstrates that no C diffusion into the γ/ γ″/γ′ microstructure has taken place. As only the γ/ γ″/γ′ microstructure and no carbide was measured inside the tip, spark spectrometry was performed to measure the overall C content. The measured value of 0.1 at% shows that a minor carbon uptake has taken place, which is in the range reported for MIM processing [7]. Since the APT measurements show very low dissolved C content, this indicates that the elevated C content is localized in discrete carbides that are visible as white phases in SEM images (Fig. 4b–d). Measured C includes atomic C well as FeC and NbC. No partitioning of C neither to the matrix nor to the γ' or the γ″ precipitates is detected. Impurity uptake in the course of debinding and sintering is generally undesirable for nickel-base superalloys. Even though grain boundary carbides resulting from carbon pick-up during binder degradation can contribute to enhanced high-temperature strength, a high volume fraction of high-temperature stable carbides can have detrimental effects on mechanical properties [19]. The APT measurements in combination with spark spectroscopy prove that the amount of solute C lies below the maximum C content allowed for IN 718 according to data sheets that are in compliance with AMS 5662 and AMS 5664 [40].

**Table 3:** Chemical composition of the overall sintered and heat-treated compound and of the γ, γ′ and γ″ phases as determined by APT

Phase	Ni	Nb	Cr	Fe	Ti	Al	Mo	Co	C	O
Overall	51.6	3.0	17.1	23.0	0.8	1.2	2.3	0.4	0.02	0.3
γ	47.9 ± 0.5	0.6 ± 0.1	20.1 ± 0.1	27.3 ± 0.4	0.2 ± 0.0	0.5 ± 0.1	2.6 ± 0.1	0.2 ± 0.0	0.01 ± 0.0	0.2 ± 0.0
γ′	70.9 ± 1.2	7.6 ± 0.5	0.4 ± 0.1	2.5 ± 0.2	6.0 ± 0.1	10.6 ± 0.8	0.6 ± 0.2	0.6 ± 0.1	0.06 ± 0.0	0.4 ± 0.1
γ″	70.2 ± 2.6	18.3 ± 1.6	1.5 ± 0.0	1.8 ± 0.0	3.5 ± 0.3	0.6 ± 0.1	2.1 ± 0.2	0.7 ± 0.1	0.04 ± 0.0	0.4 ± 0.1

The carbon measurement summarizes atomic C as well as FeC and NbC. Oxygen includes the sum of all metal oxides occurring in the atom probe measurement. These are Fe-, Al-, Nb-, Ni-, Mo-, and parts of Co-Oxides. All values in at%

Likewise, uptake of atmospheric oxygen must be minimized to avoid oxide formation, e.g., at prior particle boundaries (PPBs) [2]. The measured APT tip does not show a separate oxide phase. Present oxygen emerges from electropolishing during sample preparation as well as oxygen diffusion during heat treatments. In the course of laser APT, this oxygen is recorded as metal oxides, i.e., Fe-, Al-, Nb-, Ni-, Mo-, and parts of Co-oxides.

### Mechanical properties

Hardness of as-sintered specimens increases with increasing sintered density. A high hardness of 350 ± 45 HV_0.2_ has been measured after vacuum processing. The high hardness indicates precipitation of γ′- and γ″-phases during slow cooling after sintering [34]. Consequently, hardness is reduced to a value of 191 ± 9 HV_0.2_ by solution annealing with subsequent air cooling that prohibits the formation of hardening precipitates. After aging for 8 h at 720 °C and 8 h at 620 °C, a hardness of 446 ± 17 HV_0.2_ is achieved. These values compare well to conventional cast and forged IN 718 in identical heat-treated condition with a measured hardness of 452 ± 9 HV_0.2_. The superior hardness after aging is a clear indication for γ′ and γ″-precipitation during the heat treatment.

**Figure 7:**
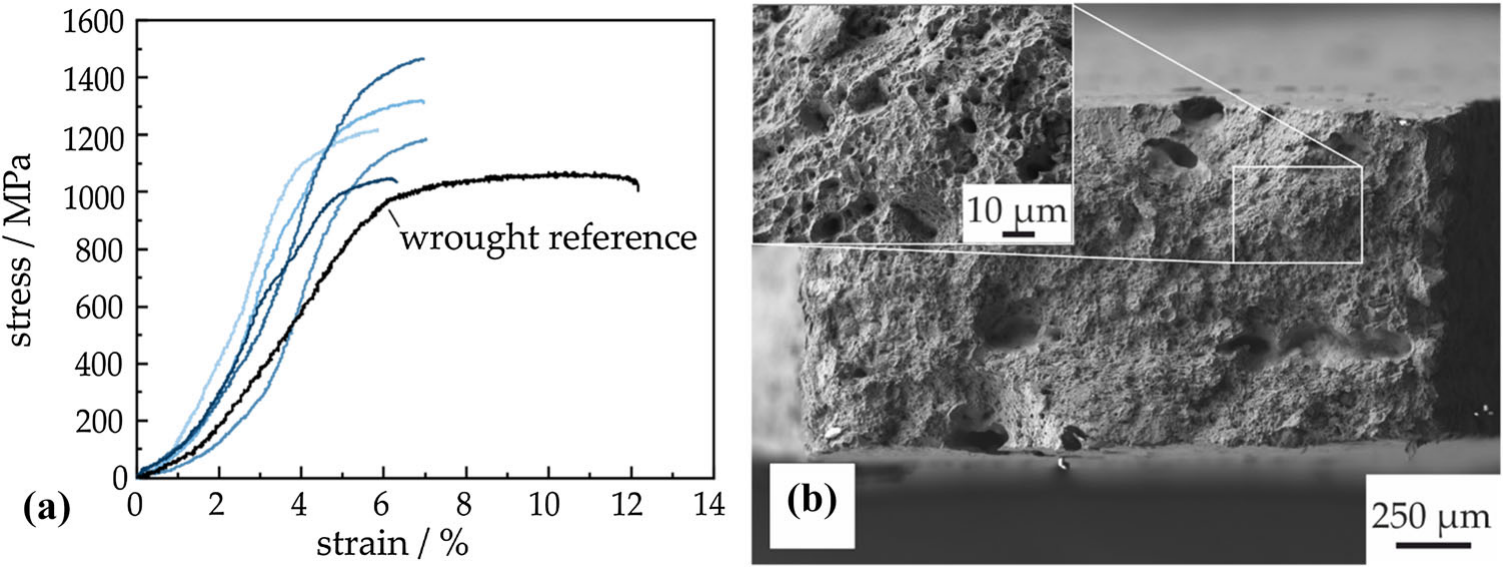
**a** Micro-tensile strength of reference (black) and representable sintered and heat-treated MF^3^ specimen (displayed in different shades of blue for visual clarity),** b** the fractured surface of MF^3^ specimen contains a few dispersed pores up to 10 µm in size. Intermediate pore free regions show dimples that are characteristics for ductile fracture

Micro-tensile testing in Fig. 7 shows high strength of the heat-treated specimen, which achieves a mean ultimate tensile strength (UTS) of 1247 ± 140 MPa. This UTS is similar to or even above MIM IN 718 [9, 16]. Conventional wrought and heat-treated IN 718 that was measured as a reference has a tensile strength of 1089 ± 62 MPa with 12.4 ± 0.8% elongation at break. One representative measurement of a wrought specimen is shown as a black curve in Fig. 7a. For visual clarity, graphs of the various MF^3^ specimens are displayed in different shades of blue. Elongation at break is relatively low in case of the MF^3^ specimens with 6.6 ± 0.5%, and almost no macroscopic ductile deformation can be observed. The brittle character of the fracture is a result of the residual porosity. The fractured surface in Fig. 7b shows that remaining pores are elongated under the applied tensile stress and the reduced cross-section is not able to withstand higher deformation. Variations in the performance of different specimens are due to random distribution of pores inside the sample volume causing an inhomogeneous stress distribution and micro-plasticity at low stresses. Despite the variations, the requirements of AMS 5917 (MIM) can be fulfilled by an optimized debinding and sintering process [5].

**Figure 8:**
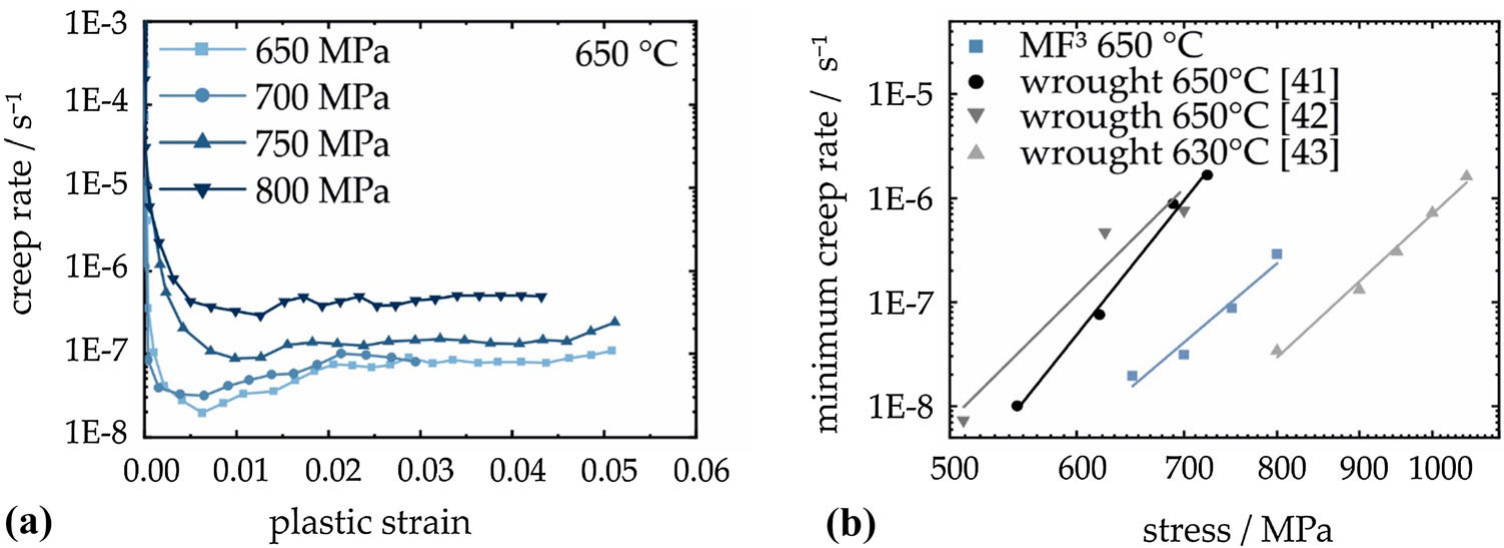
**a** Creep behavior of sintered and heat-treated MF^3^ specimen at test temperature of 650 °C and applied constant stresses of 650 MPa, 700 MPa, 750 MPa and 800 MPa as a plot of the logarithmic creep rate as a function of plastic strain.** b** Norton plot of benchmark wrought IN 718 in tensile [41, 42] and compression creep tests [43] and MF^3^ material

With respect to intended applications at elevated temperatures, the minimum creep rate of the secondary creep stage is of great importance. The compression creep behavior of heat-treated parts at 650 °C shows the expected trend of increasing minimum strain rate with higher constant stresses (Fig. 8a). Consequently, the minimum strain rate rises about one order in magnitude when increasing the stress from 700 to 800 MPa. Minimum creep rate of the additively manufactured specimen was lower than the forged and fully heat-treated state [41–43]. At a temperature of 650 °C and under the applied stresses, dislocation creep with dislocation glide and climb is expected to be the primary deformation mechanism that critically defines the lifetime of creep specimen during steady state creep [44, 45].

A comparison of the Norton plots (Fig. 8b) shows a stress exponent of 13 for the additively manufactured specimen in aged condition. This value is similar to exponents observed for forged IN 718 during tensile creep and indicates dislocation creep [41, 42, 44, 45]. Due to the large grain size after sintering and at the applied test parameters, diffusion creep along grain boundaries does not significantly contribute to creep deformation of the precipitation hardened MF^3^ IN 718. However, the slope of the linear fit is lower than of conventional material implying an improved resistance against creep damage, especially at lower stresses. This deviation from the conventional performance is a consequence of the processing history that results in a δ precipitate-free (Ni_3_Nb, D0_a_) microstructure with coarse grains and a remaining porosity up to 5%. In conventional IN 718, a small amount of plate-like δ phase can hinder grain growth at high temperatures. This task is fulfilled by grain boundary pores and grain boundary carbides in the case of the sintered specimen. The absence of δ precipitates therefore contributes to a higher availability of the γ″ (Ni_3_Nb, D0_22_) forming element Nb and thus likely to a higher volume fraction of the strengthening phase [43, 46]. For optimal creep strength, a γ″- size of 23 nm was considered beneficial [44], which was almost achieved for the aged MF^3^ IN 718 with a value of 27 ± 4 nm.

## Conclusions

Parts from IN 718 can be successfully manufactured by the MF^3^ process. Thermal debinding and sintering in vacuum atmosphere can prevent grain boundary oxidation and enhance sintering densification. Vacuum sintering at 1280 °C for 4 h achieves densities above 97% TD. As a result of the processing history, the oxygen content is slightly increased, while the amount of carbides is similar to forged IN 718. Conventional heat treatment induced the formation of γ′ and γ″ precipitates that are the important hardening phases in IN 718. Despite the remaining porosity, mechanical properties after heat treatment are comparable to those of conventionally manufactured IN 718. The hardness in aged condition reaches 447 HV_0.2_, which corresponds well to the reported value for conventional heat-treated nickel-base superalloy IN 718. High hardness after heat treatment states precipitation of γ′ and γ″-phases that contribute to high strength in micro-tensile testing. Ductility is reduced by remaining pores that are elongated under tensile load and decrease the effective cross-sectional area. Nevertheless, the mechanical requirements of the AMS 5917 standard can be fulfilled by an optimized MF^3^ process. Creep behavior similar to conventional IN 718 is achieved under compression loading.
